# Supplementary material for: “Resilience amidst challenges”: Healthcare users’ experiences of access and utilisation of primary healthcare services during the COVID-19 pandemic in southwestern Uganda
Source: PLOS Glob Public Health. 2025 Aug 18;5(8):e0005046. doi: 10.1371/journal.pgph.0005046 (PMC12360571; doi:10.1371/journal.pgph.0005046)
Supplement: S1 Text — (DOCX) [file pgph.0005046.s001.docx]

**COVID-19 HWI PROJECT QUALITATIVE INTERVIEW/DISCUSSION TOPIC GUIDE FOR COMMUNITY MEMBERS**

1. **Basic data and background**
2. Could you tell me a bit about yourself? Your background, what you do for a living?

- Age in years
- Sex
- Marital Status
- Education background
- Occupation/ work
- Facility name
- Facility type
- Village
- Duration in village
- Residential status
- Location of health facility (Urban, rural, Peri-urban)

1. Could you tell me about what a normal day looks like for you?

- Comparing BEFORE and DURING Covid 19.

1. **Ideas around illness, disease and health**
2. Could you tell me a bit about your own experience of illness?

- Common illnesses
- Frequency of sickness
- What they do when they get sick? (self-medication, clinics, health facility, traditional etc)
- What they did the last time they got sick?

1. Can you explain to me how you and your family try to stay healthy and protect yourselves from illness in daily life?

- Diet/ Feeding
- Immunization
- Antenatal Care
- Self-medication/ First Aid
- Consultation with personal physicians
- VHT intervention
- Traditional practitioners

1. If you and someone in your family becomes ill enough to seek help, what would you do?
2. *Who do you go to for help?*

- VHT
- Health workers
- Traditional healers
- Prayers

1. *How do you decide?* ***Formal and informal health management.***
2. **Experiences of COVID- 19 outbreak and response**
3. Could you tell me about your views on COVID-19 and its severity in your region?

- Probe for the signs and symptoms of COVID 19.
- How COVID is spread.
- Prevention/ Coping strategies
- Severity of COVID in comparison to others like HIV/AIDS and NCDs (severe, very severe)

1. How do you think the COVID pandemic began? What do we know, and what remains unclear?

*What do we know.*

- The countries of origin.
- How it came to Uganda
- Myths and misconceptions about COVID 19
- Treatment of COVID
- Management of COVID
- Vaccine for COVID

What remains unclear?

- Life span of virus in air and surfaces.
- Covid Deaths visa vie the spread.
- Symptomatic and asymptomatic cases
- Reinfection after vaccination/ non vaccination
- Severity of the vaccinated cases
- Question about its end or shall live with it like HIV/AIDS, flue

1. How has COVID-19 affected your community, your family and friends?

- Relationships/family interactions
- Life after work
- Stigma
- Education/School
- Gender Based Violence
- Income/ Expenditure
- Poverty
- Hunger
- Social gatherings
- Teenage/ Sexual engagements

1. Could you tell me about the health response to COVID in your region?

- Lock down (Schools, transport, cerfew, worship places, recreation places etc)
- SOPs
- COVID relief money (Nabanja’s money)
- Government giving out food
- National mask distribution
- Mass vaccination
- PPE
- Oxygen provision
- Government gazette centres into COVID centres

1. What measures have been taken, and how do you think the response has been going?

- Positive, why?
- Negative, why?

1. What do you think of the government response to COVID-19?

- Lockdown
- Vaccination
- Masks
- PPE
- Closing of boarders/ airports
- COVID relief money
- Food distribution

1. How has your community responded to COVID-19? (ie measures have been put in place/ how were messages about the pandemic received)

*Measures*

- Hand washing facilities
- Fines/ Punishments
- Community radios
- VHT sensitization
- COVID-19 Community committee
- Defense committee working hand in hand with government security personnel

*Message receipt*

- Through VHTs/ chair committee
- How was the message received/taken by the community?
- What was the people’s reaction on receipt of the message?

1. If you were in charge, what would you do to respond to the COVID-19 pandemic?

- Vaccination
- Free PPE to health workers
- Testing
- School Education
- Other health services
- Business support packages

1. How, if at all, has COVID changed your life?

- Relationships/family interactions
- Travel
- Stigma
- School/Education
- Income/ expenditure
- Social gatherings
- Poverty

1. Could you tell me about the biggest health problems facing your community?

- Accessibility to the facility
- Accessibility to the health services
- Availability of the drugs and services
- Increased co-morbidities
- Capacity of health work force
- Capacity of the health facility

1. What needs improving, and whose responsibility do think this is?

- Individual/ staff
- Community
- Government
- NGOs
- Private entities

1. How important is COVID is your own priorities when trying to stay healthy?

- COVID Vs other diseases management and treatment

1. Are there other diseases your more concerned about? Why? (*Mention them and why the concern).*
2. Have you heard any concerns or controversies about COVID in your region?

*Concerns*

- Stigma
- Overriding all other health care services (↑ co-morbidities, ↓immunizations, ↑maternal mortalities, ↓neo-natal care etc.)
- Most deaths associated with COVID-19
- Death of health care workers due to the pandemic
- Mental health of health care workers
- Reduced health workforce due to lack of PPE
- Motivation of health workers/ Remuneration
- Family/partner/ relationship concerns

*Controversies*

- COVID-19 is not real/ Myth
- Witchcraft
- Misconception about COVID-19 vaccine (*its efficiency and assumed eventual death*)
- Political agenda
- Climate Vs the survival of COVID-19
- Africans resistant to COVID-19

1. IF so, why do you think people have these fears? Do you agree or disagree, and why?

- Consider the origin of the fears in relation to COVID 19.
- New pandemic and little know about it.
- Myths and misconceptions about COVID
- Government looking at increasing numbers for financial benefit.

1. **Experiences and healthcare in COVID**
2. Have you needed to access primary health care services at your local health facility since the COVID pandemic began? Antenatal care? Out-patient services? Family planning? Immunization services or HIV/AIDS services? *Probe for more services.*
3. When did you need to access services (early or late in the pandemic)? Two pandemics *(1-early March-July 2020, late September, 2-early June 2021- July, late- on going)*
4. Did you go ahead and access services? Could you tell me about your experience and challenges of accessing primary health care before and during the COVID outbreak? *Probe for the services talked about earlier. Probe for reasons if answer is yes or no.*
5. In your opinion, has the COVID outbreak had an impact on primary health services in your region? If so, in what way?

- Positive, why?
- Negative, why?

1. Has it presented any challenges in your ability to access services? *Yes or no, why?*
2. Since the COVID-19 outbreak, have you used primary healthcare services in the same way as you did before? *If so, why? If not, why not?*
3. Could you tell me about any concerns you might have had about accessing healthcare services during the COVID outbreak?

- Equipment and medical supplies at facility
- Health workforce (*numbers, attitude, training, etc*)
- Accessibility to the health facility (*transportation, restrictions, openness of the facility etc*)

1. Do you think that COVID-19 has increased the costs involved in seeking healthcare in your catchment area?

- Have transport costs increased?
- Have there been issues with drugs supply etc?) [If yes, ask for a breakdown of added costs]

1. Have you noticed any changes in your family or community members’ access to healthcare? Are you aware of any concerns they might have?

- What changes happened and why they happened?
- Probe for concerns they might have.

1. In your experience, what are the challenges to accessing primary health care at the moment? *Probe in relation to health care giver, health care user, health care facility and the community.*

- Lack of funds
- Fears for contracting infection
- Accessibility challenges (distance, transport restrictions etc)
- Health facility challenges (workforce, PPE, facility capacity etc)

1. Have you noticed any change in the provision of healthcare since the COVID outbreak? If so, what changes have you seen? What do you think has caused these changes?

- *Probe in relation to health care giver, health care user, health care facility and the community.*

1. **Health seeking behavior and trust**
2. What options are there for Health services in your community?

- Available health facilities (*Government, private entities and nature of facility or level*)
- Type of services accessed eg community outreaches.

1. Is there anyone who you would not trust for healthcare and help, and if so, why not?

- Access to professionals or non-professionals.

1. How would you describe your experience with healthcare workers and health facilities?

- Positive or negative, why?

1. Could you tell me about the biggest health problems facing your community? What needs improving and whose responsibility do think that is?
2. In your opinion, what is the most reliable source of information on health and disease? How do you decide which information to trust? (*Personal take on the trust and why*?)

- TV, radio, new papers
- Social media i.e. face book, WhatsApp
- Local sources ie LC audio sources and community leaders, peers
- Health workers ie doctors, clinical officers, nurses, nursing assistants
- VHTs
- Researchers

1. Where have you accessed information about the COVID-19 pandemic? What has your impression been about the reliability of this information?

- Probe on the sources.
- Do you trust the information and why or why not?

1. Have you heard any discussions around medical research or vaccine trials in your country?
   - If so, what are people saying?
   - Have you heard any concerns? Where do you think they come from?
   - How do you feel about these concerns?
   - What impact do you think these concerns have on trust in health services?
2. What do you think about the future possibility of COVID-19 vaccines in your country?
3. What role do you think COVID-19 vaccination should or could play in responding to the pandemic in your country?
   - what do you think might be some opportunities and challenges involved in vaccinating people against COVID-19 in your country? If so, could you tell me more about these?
