# Supplementary material for: “Resilience amidst challenges”: Healthcare users’ experiences of access and utilisation of primary healthcare services during the COVID-19 pandemic in southwestern Uganda
Source: PLOS Glob Public Health. 2025 Aug 18;5(8):e0005046. doi: 10.1371/journal.pgph.0005046 (PMC12360571; doi:10.1371/journal.pgph.0005046)
Supplement: S2 Text — (DOC) [file pgph.0005046.s002.doc]

**COVID-19 HWI PROJECT CODE BOOK- HEALTH CARE USERS**

**Codes**

| **Name** | **Description** |
| --- | --- |
| **Community Health Challenges** | **This relates to the greatest challenges faced by the community for primary health care delivery.** |
| COVID 19 related | This talks about challenges that are related to or have been caused by COVID-19 |
| Not COVID 19 related | This talks about challenges that are not related to the pandemic or existed even before COVID-19; individual, systemic, infrastructure or health facility related etc. |
| **Community Perceptions of Healthcare Utilization during COVID-19** | **This looks at the general community perceptions about health care usage during the pandemic.** |
| Attitudes | This looks at the general attitudes, positive or negative, of the community members concerning the health services. |
| Beliefs | This looks at beliefs, theories and opinions about illness and disease, including COVID-19. |
| Expectations | This is about what people expect from the health care system; facility and health workers. |
| Risk Perceptions | This explores that options available and perceived risks for seeking healthcare at the health facilities. |
| **Consent** | **This describes the consenting process.** |
| **Health Seeking Behaviour and Trust** | **This talks about what community members do when they are ill and what they do to keep healthy. It describes the trust or mistrust people have in the health care system or facility.** |
| Challenges | This talks about the challenges that community members face while trying to seek health care services. |
| Concerns and controversies | This describes the concerns, controversies, myths and misconceptions and what remains unclear about the COVID-19 pandemic; and how such affect the conduct of community members. |
| COVID 19 vaccination and testing | This explores people's perceptions about the COVID-19 testing, the COVID-19 vaccine and the vaccination process, and the uptake. |
| Impact on trust in health services | This explains the impact of the COVID-19 testing and vaccination on the trust that community members attach to health care service provision. |
| Knowledge | This explains what community members know about the COVID-19 disease; the causes, its origin and effects. |
| Opportunities | This explores the opportunities that could arise as result of COVID-19 testing and vaccination. |
| **Ideas around illness, disease and health** | **This describes the experiences of illnesses other than COVID 19.** |
| COVID 19 knowledge | This is about what the community members know about COVID-19. |
| Health Seeking behaviour | This is about how people go about getting treatment for their illness, be it formal or informal (traditional). It also relates to getting treatment from government health facilities, clinics, personal prescriptions, traditional medicine and traditional healers. |
| Knowledge of illness | This is about if/ what people know the diseases or illness that are most common in their communities and what causes them and their effects too. |
| **Impact of COVID 19** | **This elaborates the general impacts of the COVID-19 pandemic.** |
| Cost of seeking health care | This explores the impact of COVID 19 on the costs involved in seeking health care and accessing healthcare facilities e.g. monetary, infrastructural, transportation etc. |
| Delivery of Health Services | This elaborates the impact of COVID-19 on the delivery of health care services in general. |
| Personal life | This explores the general impact of COVID-19 at a personal level; family, health, social life, business etc. |
| Professional Life | This describes the impact of COVID-19 pandemic on the professional life of community members and changes in their day-to-day lives. |
| Social Networks | This relates to the social networks people had during COVID-19 and how influential they were in coping with the pandemic. |
| Stigma and mental health | This describes the experiences of stigma of victims from family, friends and the community at large and the effect this stigma and the actual pandemic had on their mental health. |
| Trust and Attitudes towards Health Services | This looks at the impact of COVID-19 on trust and attitudes of community members toward health care services in general. |
| **Institutional Changes during COVID 19** | **This relates to the actual changes at the health facility, be it structural changes, health workers numbers, length of time spent at the clinic and with a patient, admissions etc.** |
| **Observations** | **This describes the observation made by the interviewers at the health facilities on the day of the interview.** |
| **Personal Experiences of COVID 19 outbreak and responses** | **This highlights the firsthand experiences of health care users and their various responses towards the outbreak.** |
| Health Care Users | This talks about the experiences and responses of the community members; how their livelihoods, treatment seeking, families, social lives, school, businesses etc. were affected and how they coped with such challenges. |
| **Responses to COVID 19** | **This explores the responses to the COVID-19 pandemic by the government and communities** |
| Challenges | This explains the challenges involved in implementing the COVID-19 measures. |
| Community | This looks at the measures put in place by the community to respond to COVID-19. |
| Government | This is about the responses that were put in place by the government to respond to the COVID-19 pandemic. |
| Individual level | This relates to what the community member do to respond to the COVID 19 pandemic if they were in charge. |
| **Socio-demographics** | **This refers to the basic data and background information about the participants/ community members.** |
| **Sources of Information on Health and Disease** | **This node talks about the different sources of information about health and diseases, COVID-19 inclusive** |
| Community | This explains where the community members generally get information about health care and diseases. It also relates to the source of information about COVID-19 and how to prevent against it. |
| **Suggestions and Recommendations by participants** | **This looks at what needs improving in the health care system and whose responsibility it is.** |
| Healthcare Users | This is about the suggestions and recommendations of the community members on how the health care system can be improved during the COVID-19 pandemic and whose responsibility it is. |
| **Work** | **This explains what a normal day looks like for health users.** |
| Before COVID 19 | This talks about how a normal day looked like for both the community members/ health users, before the COVID-19 pandemic. |
| Changing perceptions about work | This is about the changing perception about work for the health users; the change in actual work done, additional roles, workload, loss of work and jobs. |
| During COVID 19 | This talks about how a normal day looks like during the COVID-19 pandemic for the health care users |
