# Supplementary material for: “Resilience amidst challenges”: Healthcare users’ experiences of access and utilisation of primary healthcare services during the COVID-19 pandemic in southwestern Uganda
Source: PLOS Glob Public Health. 2025 Aug 18;5(8):e0005046. doi: 10.1371/journal.pgph.0005046 (PMC12360571; doi:10.1371/journal.pgph.0005046)
Supplement: Attachment — Submitted filename: Responses to reviewers.docx [file pgph.0005046.s004.docx]

25th July 2025

**Response to Reviewers**

Dear Editor (Ferdinand C Mukumbang),

We are pleased that PLOS Global Public Health journal has given our manuscript the opportunity to be reviewed and will consider it further for publication after we have addressed all comments raised by the reviewers and editor. Below is a point-by-point explanation of how we have addressed the concerns and revised our manuscript. We have included the manuscript section, page and line number to show where we have made track changes representing each response. The page and line numbers which we refer to are from the tracked changes version.

Sincerely,

Rachel Kawuma (corresponding author)

1)Provide a more detailed justification for the selection of the 15 health centres.

**Response:** We have included a detailed justification why the 15 health centres were selected in the sampling and recruitment section, on page 6, lines112-116 and 117-123 of the tracked changes version to read as follows:

Fifteen out of 25 health facilities were selected to ensure variation in community contexts, facility levels, and geographical settings within Masaka District. Facilities with significant service disruptions were prioritized to understand how the pandemic affected care provision and access. The mix of facility levels (HC II, III, and IV) captured differences in capacity, infrastructure, and services.

The inclusion of both urban and rural health facilities explored how location influenced healthcare users' experiences and community health care access during the pandemic, notwithstanding how the different socio-economic and geographic realities affected access to care. Given logistical and safety constraints during the pandemic, the selected facilities were those that were logistically feasible to reach by the research team within the study period, while still maintaining health protocols and representation across the district.

2) Expand on the description of the data analysis process, including details about the coding process and inter-coder reliability.

**Response:** We have expounded on the data analysis process to describe discussions that ensured that there was inter-coder reliability on page 8, lines 179-181 of the data management and analysis section and it reads as follows:

To increase inter-coder reliability and validity, regular de-brief meetings were conducted to discuss coding decisions on the same data segments, clarify understanding of the codes and refine their interpretations.

3) Elaborate on the potential impact of social desirability bias.

**Response:** We have included an explanation in the strength and limitations section, lines 538-543 to read as follows:

Potential for social desirability bias in participant responses may have stemmed from fear of losing access to health services when discussing challenges faced at the health facilities. Particularly because interviews were conducted at the health facilities where health workers could see them. Additionally, incarcerated participants may have feared retribution from prison authorities. To address this, we included probing and consistency check questions to help verify the reliability of participants responses.

4) Clarify why healthcare workers were not included in the analysis.

**Response:** The project aimed to examine how, and to what extent, the COVID-19 pandemic affected the provision and utilisation of primary health care services. It had five objectives, including documenting community members’ experiences in accessing primary health care during the outbreak and identifying the barriers and facilitators to its utilisation. Another objective, which we hope to address in another manuscript was to document the experiences of primary health care workers in delivering care during the outbreak, along with the barriers and facilitators they encountered. We have included this under the study setting and population section, on page 5, lines 106-112.

5) Reformat Table 2 to improve readability.

**Response:** Thank you for this observation, table 2 has been reformatted and is on page 11 of the manuscript.
